# Shunt Surgery Efficacy Is Correlated With Baseline Cerebrum Perfusion in Idiopathic Normal Pressure Hydrocephalus: A 3D Pulsed Arterial-Spin Labeling Study

**DOI:** 10.3389/fnagi.2022.797803

**Published:** 2022-02-23

**Authors:** Wenjun Huang, Xuhao Fang, Shihong Li, Renling Mao, Chuntao Ye, Wei Liu, Guangwu Lin

**Affiliations:** ^1^Department of Radiology, Huadong Hospital Affiliated to Fudan University, Shanghai, China; ^2^Department of Neurosurgery, Huadong Hospital Affiliated to Fudan University, Shanghai, China

**Keywords:** idiopathic normal pressure hydrocephalus (INPH), pulsed arterial-spin labeling (PASL), neuroimaging, dementia, gait disorder, incontinence

## Abstract

This study investigated the relationship between preoperative cerebral blood flow (CBF) in patients with idiopathic normal pressure hydrocephalus (INPH) and preoperative clinical symptoms and changes of clinical symptoms after shunt surgery. A total of 32 patients with diagnosed INPH and 18 age-matched healthy controls (HCs) were involved in this study. All subjects underwent magnetic resonance imaging (MRI), including 3D pulsed arterial-spin labeling (PASL) for non-invasive perfusion imaging, and clinical symptom evaluation at baseline, and all patients with INPH were reexamined with clinical tests 1 month postoperatively. Patients with INPH had significantly lower whole-brain CBF than HCs, with the most significant differences in the high convexity, temporal lobe, precuneus, and thalamus. At baseline, there was a significant correlation between the CBF in the middle frontal gyrus, calcarine, inferior and middle temporal gyrus, thalamus, and posterior cingulate gyrus and poor gait manifestation. After shunting, improvements were negatively correlated with preoperative perfusion in the inferior parietal gyrus, inferior occipital gyrus, and middle temporal gyrus. Preoperative CBF in the middle frontal gyrus was positively correlated with the severity of preoperative cognitive impairment and negatively correlated with the change of postoperative MMSE score. There was a moderate positive correlation between anterior cingulate hypoperfusion and improved postoperative urination. Our study revealed that widely distributed and intercorrelated cortical and subcortical pathways are involved in the development of INPH symptoms, and preoperative CBF may be correlative to short-term shunt outcomes.

## Introduction

Normal-pressure hydrocephalus (NPH) is a hydrocephalus syndrome characterized by the clinical triad of gait disturbance, cognitive decline, and incontinence, with ventricular enlargement and a normal cerebrospinal fluid (CSF) pressure. Clinically, it can be classified as idiopathic normal pressure hydrocephalus (INPH) or secondary normal pressure hydrocephalus (SNPH) based on whether the etiology is definite (Adams et al., 1965). At present, confirmation of INPH depends on the CSF tap test and shunt surgery (Nakajima et al., 2021), and clinical symptoms are treatable by permanent drainage (Relkin et al., 2005). However, a large number of patients with INPH are complicated with comorbidities, such as Alzheimer’s disease (AD), Parkinson’s-like diseases, extrapyramidal dyskinesia diseases (Allali et al., 2018), or similarities with normal aging (Agerskov et al., 2018), which may be the reason for a poor or even negatively impacting shunt response (Broggi et al., 2016; Bräutigam et al., 2019; Macki et al., 2020). With up to 40% of patients not responding, it remains difficult and essential to select patients who would benefit from surgery (Giordan et al., 2018). Therefore, by analyzing the relationship between baseline cerebral perfusion and changes in clinical symptoms after shunt, we attempted to infer the neural substrates underlying changes in clinical manifestations of INPH in order to screen the shunt surgery recipients and minimize unnecessary invasive procedures.

There is evidence that the pathological mechanism of INPH is complex and not only results from CSF circulation disorder (Lalou et al., 2018) but is also related to cerebrovascular self-regulation disorder and abnormal brain metabolism (Landau et al., 2014). Most studies have shown a significant decrease in regional cerebral blood flow (rCBF) in patients with INPH compared with healthy elderly individuals (Ziegelitz et al., 2014; Virhammar et al., 2017; Mattoli et al., 2020). Most studies have demonstrated that whole and regional cerebral blood flow (CBF) in patients with INPH were significantly lower than those in normal controls. Existing studies have not been consistent with the relationship between baseline CBF and change in clinical symptoms after shunt surgery. White matter CBF has been shown to gradually increase with greater distance from the ventricle, especially the lateral ventricle. In terms of gray matter, the frontal cortex and central gray matter were predominantly affected (Virhammar et al., 2014; Ziegelitz et al., 2016; Tuniz et al., 2017; Mattoli et al., 2020). Some studies suggest that hippocampal perfusion also decreased (Ziegelitz et al., 2014; Ziegelitz et al., 2015).

Arterial spin labeling (ASL) is a relatively new non-invasive perfusion imaging method that utilizes endogenous blood-based water molecules as tracers (without ionizing radiation) to visualize and quantify CBF (Jezzard et al., 2018). Based on the persistent stability of internal brain metabolic activity in the resting state, ASL can explore and monitor alterations in tissue perfusion in states of brain dysfunction (Soldozy et al., 2019).

The study aimed to determine the relationship between baseline CBF and preoperative clinical symptoms and the efficacy of shunt surgery. We hypothesized that baseline CBF may be associated with clinical changes after shunt surgery and help screen patients with INPH for shunt surgery clinically.

## Materials and Methods

### Subjects

This study was approved by the Institutional Review Board of Huadong Hospital affiliated with Fudan University (approval number: 2017K027). The ethics committee waived the requirement of written informed consent for participation.

We retrospectively reviewed patients with INPH who were admitted to the inpatient unit at the neurosurgery department of Huadong Hospital affiliated with Fudan University to undergo shunt surgery from May 2019 to July 2021. The inclusion criterion for patients with diagnosed INPH according to expert consensus on the diagnosis and treatment of INPH in Experts consensus on diagnosis and treatment of normal pressure hydrocephalus in China (2016) were as follows: (1) age over 60 years; (2) the presence of at least one of the triad of symptoms (i.e., gait disturbance, dementia, or incontinence) with insidious progression for more than 6 months; (3) ventricular dilatation (Evans’ index > 0.3); (4) CSF pressure < 200 mm H_2_O; (5) the absence of other diseases that might account for such symptoms; and (6) underwent magnetic resonance examination, CSF tap test, and lumboperitoneal shunt surgery. The exclusion criteria for the patients with INPH were as follows: (1) cerebral infarction and dementia caused by clear causes and hospitalization for severe mental illness and (2) SNPH.

The inclusion criteria for elderly healthy controls (HCs) were as follows: (1) age over 60 years; (2) no gait disorder, cognitive impairment, or urination disorder, and normal Mini-Mental State Examination (MMSE) score; (3) conventional cerebral magnetic resonance imaging (MRI) showing no abnormalities; and (4) no active neurological, systemic, or psychiatric diseases.

Trained neurologists performed the clinical examinations. Besides a standard neurological examination, the tests included the INPH grading scale (INPHGS), MMSE, and for the patients with INPH, the timed up and go test (TUG-t) (Nakajima et al., 2021), before and 1 month after shunt surgery. For the INPHGS, motion disturbance, cognitive impairment, and incontinence are rated from 0 to 4. The higher the score was, the more severe the symptoms.

**FIGURE 1:**
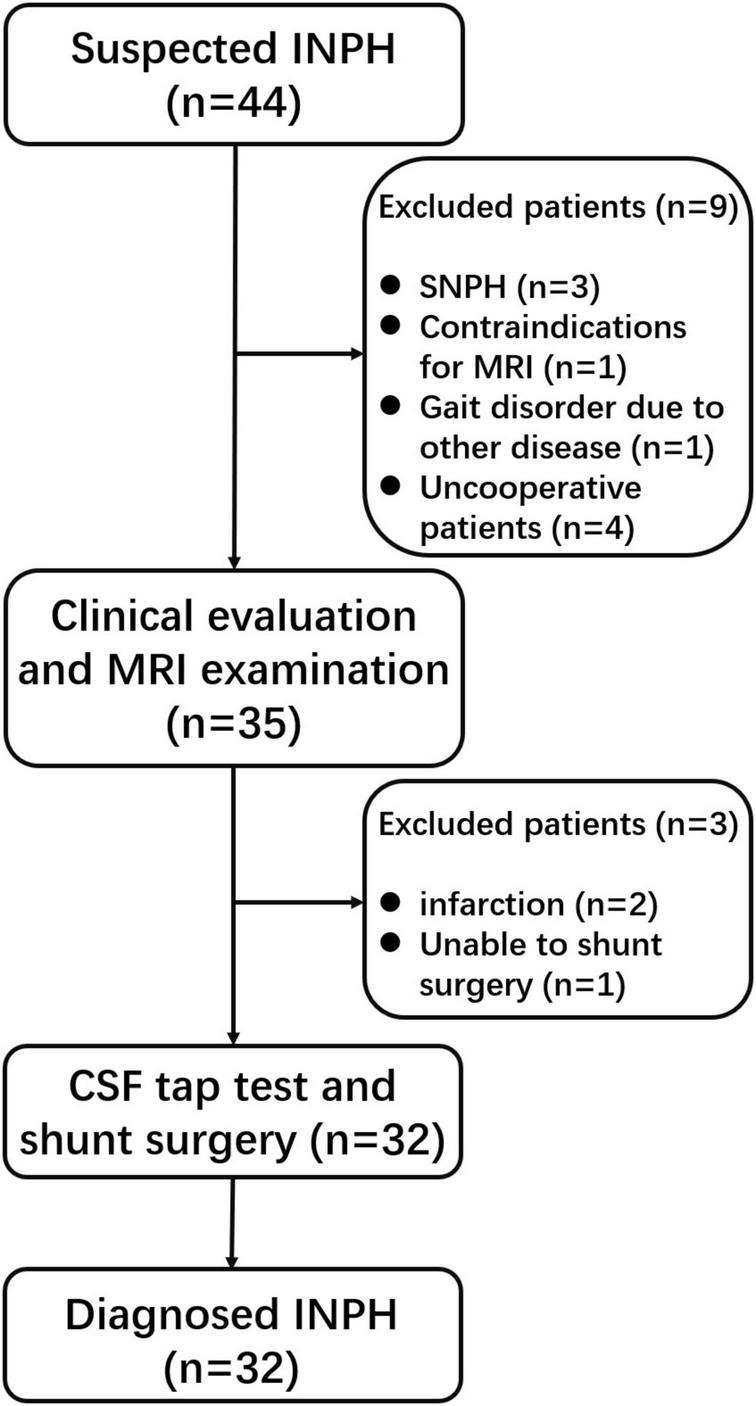
Flowchart describing the inclusion process of patients.

In total, we enrolled 32 patients with diagnosed INPH and 18 HCs into the study. The flowchart of enrollment of the diagnosed patients with INPH is shown in Figure 1. Demographic data and clinical characteristics are shown in Table 1.

**TABLE 1:** Demographic and clinical data in the diagnosed INPHs and HCs.

	INPH (*n* = 32)		Healthy control, HC (*n* = 18)	*p1*-value	*p2*-value
	Preoperative	1 month postoperative		Pre vs. HC	Pre vs. post
Age (average, range)	75.22 (66∼89)		72 (63∼82)	0.185	
Gender (male/female)	24/8		6/12	0.032*	
**INPHGS**					
Motion	3 (0)	2 (1)	0 (0.25)	< 0.001**	< 0.001**
Cognition	3 (1)	2 (0.75)	0 (1)	< 0.001**	< 0.001**
Urination	2.5 (1)	1 (1)	0 (0)	< 0.001**	< 0.001**
Total	8 (2)	5 (1)	0 (1.25)	< 0.001**	< 0.001**
MMSE	17.5 (8)	23 (7)	29 (2)	< 0.001**	0.037*
TUG-t	20.15 (10.09)	15.86 (3.85)	9.93 (1.58)	< 0.001**	0.001**

*Values denote the median (quartile).*

Significant differences are marked with *p<0.05 and **p<0.01.

### Magnetic Resonance Protocol

All magnetic resonance (MR) data were acquired using a 3.0-T MRI scanner (MAGNETOM Prisma, Siemens Healthcare, Erlangen, Germany). The imaging protocol consisted of resting-state perfusion imaging assessment *via* a 3D pulsed arterial-spin labeling (PASL) sequence and anatomical assessment *via* T1-weighted magnetization prepared gradient-echo sequence (MPRAGE). A routine clinical T2-weighted structural MRI was also acquired for the neuroradiological assessment of the participants. For this study and based on preregistration, only the PASL and the MPRAGE sequences were included in the analyses.

The following parameters were used for the PASL acquisition: scan duration: 296 s; repetition time (TR): 4,600 ms; time to echo (TE): 16.18 ms; label time: 700 ms; post-labeling delay: 1,290 ms; inversion time (TI): 1,990 ms; field of view (FOV): 192 × 192; 3 mm × 3 mm × 3 mm; slice thickness: 3 mm with a 1.5 mm gap; 40 axial slices; and the number of excitations = 4.

The following parameters were used for the MPRAGE sequences: scan duration = 3 min 39 s; TR: 1,800 ms; TE: 2.37 ms; FOV: 250 × 250; 0.87 mm × 0.85 mm × 0.85 mm; slice thickness: 0.85 mm with no gap; and 208 slices.

### Magnetic Resonance Imaging Data Preprocessing

The PASL data were processed using the SPM12 software^1^ and the ASL toolbox (ASLtbx^2^) (Wang et al., 2008). The center of each volume was first reset to the origin, and all rotations were set to zero. The first PASL image was set as the reference volume, and all other images were then motion-corrected relative to the reference. PASL images were realigned relative to the T1-weighted images for each subject. Smoothing of the realigned and coregistered PASL images was performed by applying an SPM Gaussian smoothing kernel of 6 mm × 6 mm × 6 mm full-width at half maximum (FWHM). A mask based on the mean of the smoothed PASL images was employed to exclude out-of-brain voxels. An ASL difference image was calculated using a single-compartment model (Buxton et al., 1998) after subtracting the label image from the control image. The four ASL difference images were averaged to calculate the CBF maps in combination with the proton-density-weighted reference images (Xu et al., 2010). Normalization, using T1 image unified segmentation with bounding box [–90, –126, –72; 93, 93, 111] and isotropic voxel size [3, 3, 3], could transform CBF maps to reduce the variability between individuals and allow meaningful group analyses. A quality check was performed visually to ensure the good quality of the preprocessing.

### Statistical Analysis

Clinical symptom statistics are expressed as the median (quartile). We compared the data between the preoperative INPHs and HCs, and the measurements were made preoperatively and 1 month postoperatively using the Mann-Whitney *U-*test. The statistical analysis was conducted using the Statistical Package for Social Science version 24.0 (IBM SPSS).

Cerebral blood flow maps were statistically analyzed using second-level statistical procedures as implemented in SPM12 based on a generalized linear model (GLM). The CBF in the diagnosed INPH and HC groups was compared using a two-sample *t*-test. Multiple regression was used to analyze the correlation between preoperative CBF and preoperative and postoperative clinical changes by regressing out the *z*-transformed correlation coefficients.

Gender was included as covariates in the regression. The *p*-value *p* < 0.05 [false discovery rate (FDR) corrected, using the SPM12 software (see text footnote 1)] was considered significant. CBF clusters were visualized using the xjview^3^ toolbox. Clusters with significant differences and significant correlations are displayed in pseudocolor on the calibrated standard brain map, and their Montreal Neurological Institute (MNI) coordinates and voxel sizes of peak intensity are listed in a table.

## Results

### Demographic and Clinical Data

Table 1 shows the demographic and clinical variables of the patients with diagnosed INPH and HCs. No significant differences in age were observed between the patients with INPH and HCs (*p* > 0.05). As there were significant differences between the genders, we regressed it as a covariable in all statistical analyses.

The INPHGS, TUG, and MMSE scores markedly differed between the patients and HCs (*p* < 0.001). Furthermore, all patients with INPH improved in clinical symptoms to varying degrees after shunt surgery (*p* < 0.05).

### Group Differences in Cerebral Blood Flow

**FIGURE 2:**
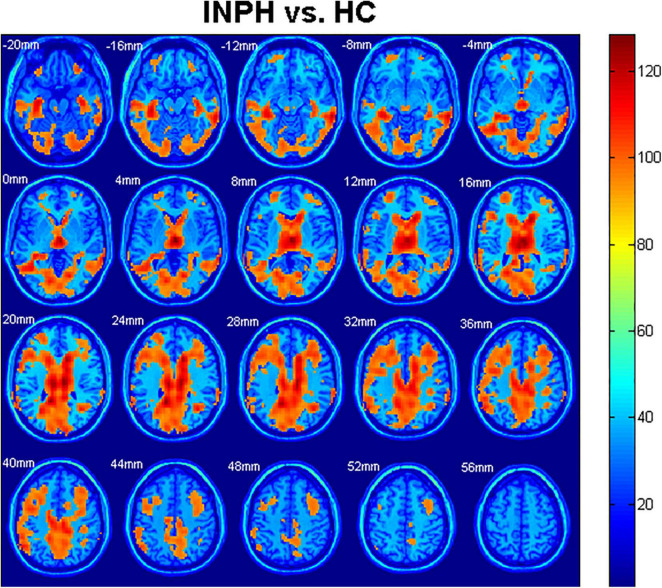
Comparison of cerebral blood flow (CBF) among idiopathic normal pressure hydrocephalus (INPH) and healthy control (HC) groups. Significant region (false discovery rate corrected *p* < 0.05) illustrated in warm colors for increased values and in cool colors for decreased values. Significant differences were revealed in the following brain regions: bilateral middle frontal gyrus (Frontal_Mid_L, R), bilateral thalamus, bilateral middle temporal gyrus (Temporal_Mid_R, L), bilateral precuneus (Precuneus_R, L), bilateral calcarine (Calcarine_L, R), bilateral inferior temporal gyrus (Temporal_Inf_L, R), corpus callosum, bilateral caudate, and bilateral middle cingulate gyrus (Cingulum_Mid_R, L) (*p* < 0.05).

Statistical analyses were observed regarding an automated anatomical atlas (AAL) template (Ashburner, 2007). The two-sample *t*-test revealed the significant differences between the patients with diagnosed INPH and HCs in the bilateral cerebrum. Generally, global and bilateral CBF was significantly lower in the INPHs than in the HCs, and the following brain areas were predominant: middle frontal gyrus (Frontal_Mid_L, R), thalamus, middle temporal gyrus (Temporal_Mid_R, L), precuneus (Precuneus_R, L), calcarine (Calcarine_L, R), inferior temporal gyrus (Temporal_Inf_L, R), corpus callosum, caudate, and middle cingulate gyrus (Cingulum_Mid_R, L) (Figure 2). The results were corrected by the FDR with a voxel-level *p* < 0.05.

### Correlative Analysis

**FIGURE 3:**
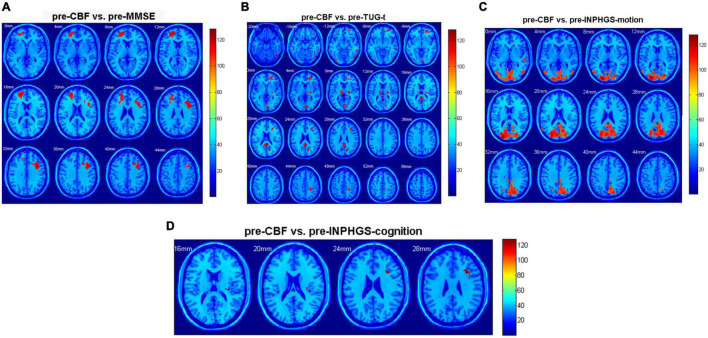
Correlation of cerebral blood flow (CBF) between brain regions and clinical scale scores in patients with idiopathic normal pressure hydrocephalus (INPH): **(A)** Preoperative Mini-Mental State Examination (MMSE) score; **(B)** Preoperative timed up and go test (TUG-t) score; **(C)** Preoperative INPH grading scale (INPHGS) motion score; and **(D)** Preoperative INPHGS cognition score.

Regarding the diagnosed INPH group, we calculated Spearman correlation coefficients between the CBF values and preoperative clinical scale scores, including the INPHGS (motion, cognition, and urination), MMSE, and TUG-t. The brain regions related to the above clinical data are indicated in Figures 3A–D and Table 2. A positive correlation was identified between the following pairs: CBF values in the Frontal_Mid_L, R, Frontal_Sup_L, and Frontal_Inf_Oper_R and preoperative MMSE scores (Figure 3A). Negative correlations were identified between the following pairs: CBF values in the Frontal_Mid_R, Calcarine_R, L, Thalamus_R, L, and Cingulum_Post_L, R and preoperative TUG-t scores (Figure 3B), CBF values of the Occipital_Mid_L, Calcarine_L, Temporal_Inf_R, Temporal_Mid_R, and preoperative INPHGS-motion scores (Figure 3C), CBF values in the Frontal_Inf_Tri_R and preoperative INPHGS-cognition scores (Figure 3D) (*p* < 0.001).

**TABLE 2:** Correlation of cerebral blood flow (CBF) between brain regions and preoperative clinical scale score in idiopathic normal pressure hydrocephalus (INPH).

Preoperative clinical scale	No.	Brain region	Cluster size	Peak MNI coordinates (x, y, z)	Peak intensity
MMSE	1	Frontal_Mid_L	380	(−15, 54, 12)	4.71
		Frontal_Sup_L			
	2	Frontal_Mid_R	300	(39, 9, 33)	5.18
		Frontal_Inf_Oper_R			
TUG-t	1	Frontal_Mid_R	125	(18, 51, −6)	3.94
	2	Calcarine_R	87	(3, −87, 3)	4.11
		Calcarine_L			
	3	Thalamus_L	45	(−9, −24, 9)	3.93
	4	Thalamus_R	61	(24, −18, 9)	4.83
	5	Cingulum_Post_L	120	(3, −45, 21)	4.69
		Cingulum_Post_R			
INPHGS-motion	1	Occipital_Mid_L	4145	(−21, −93, 12)	6.41
		Calcarine_L			
	2	Temporal_Inf_R	344	(54, −27, −3)	5.38
		Temporal_Mid_R			
INPHGS-cognition	1	Frontal_Inf_Tri_R	21	(33, 18, 27)	3.79

Furthermore, we also calculated the correlation between the CBF values and postoperative clinical score changes, including the changes in INPHGS (motion, cognition, and urination), MMSE, and TUG-t scores. The brain regions related to the above clinical data are indicated in Table 3. Positive correlations were identified between the following pairs: CBF values in the Cingulum_Ant_R and postoperative INPHGS-urination score change (Table 3) and CBF values in the Temporal_Mid_R, L and postoperative TUG-t score change (Table 3). Negative correlations were identified between the following pairs: CBF values in the Frontal_Mid_R, L, Temporal_Mid_R, caudate, and thalamus and postoperative MMSE score change (Table 3) and CBF values in the Occipital_Inf_L, Parietal_Inf_L, Precuneus_R, and postoperative INPHGS-motion scores (Table 3) (*p* < 0.001).

**TABLE 3:** Correlation of cerebral blood flow (CBF) between brain regions and postoperative clinical score changes in idiopathic normal pressure hydrocephalus (INPH).

Postoperative clinical score change	No.	Brain region	Cluster size	Peak MNI coordinates (x, y, z)	Peak intensity
MMSE	1	Frontal_Mid_R	49	(36, 21, 42)	3.83
	2	Caudate_R	68	(6, −3, 6)	4.53
		Thalamus_R			
	3	Temporal_Mid_R	36	(45, −48, 15)	3.77
	4	Frontal_Mid_L	41	(−30, 9, 42)	4.35
TUG-t	1	Temporal_Mid_R	48	(54, −24, −6)	4.49
	2	Temporal_Mid_L	13	(−54, −39, 3)	3.61
INPHGS-motion	1	Occipital_Inf_L	70	(−21, −87, −3)	4.42
	2	Parietal_Inf_L	101	(−30, −60, 42)	4.02
	3	Precuneus_R	45	(3, −72, 45)	4.09
INPHGS-urination	1	Cingulum_Ant_R	16	(6, 33, 21)	3.73

## Discussion

In this study, PASL, a non-invasive perfusion imaging technique, was used to compare cerebral perfusion in patients with INPH with that in healthy older individuals. We found that the whole-brain CBF of patients with INPH was significantly reduced, which was consistent with the results of previous studies (Virhammar et al., 2014; Ziegelitz et al., 2016; Mattoli et al., 2020), suggesting a significant decrease in the cerebral perfusion rate. Hypoperfusion of the brain could further lead to pathophysiological changes in brain tissue, particularly oxygen metabolism (including oxygen utilization and extraction) (Göttler et al., 2019).

Overall, significantly reduced CBF in the INPH group was found in the high convexity gray matter. According to the *Guidelines for Management of Idiopathic Normal Pressure Hydrocephalus* (Third Edition), disproportionately enlarged subarachnoid space hydrocephalus (DESH) can be observed in most patients with INPH, which may be a reliable imaging feature of subarachnoid CSF absorption disorder (Nakajima et al., 2021). Reduced perfusion in the high convexity brain may result in local vascular compression due to its greater compression than in other regions of the brain. In addition, the CSF in the narrow sulcus is significantly reduced compared with other parts, so the metabolic efficiency of the local brain tissue is decreased.

There was reduced perfusion in the temporal lobe. One possible reason is the temporal lobe compression due to the temporal horn dilation, and another potential cause may be the AD comorbidity, which may also explain the precuneus hypoperfusion observed in this study (Ishii, 2020). Consistent with the findings of Ziegelitz’s two studies on regional cerebral perfusion (Ziegelitz et al., 2014; Ziegelitz et al., 2015), CBF in the frontal lobe and periventricular white matter in patients with INPH was significantly lower than that in controls. Combined with previous studies (Ziegelitz et al., 2014; Virhammar et al., 2017), we speculated that the edema in the paraventricular white matter led to local compression of small vessels and metabolic disorder of vasoactive metabolites. This study found that thalamic CBF significantly decreased, which may be due to the decrease in *N*-acetyl-aspartate levels in the thalamus of patients with INPH due to impaired local metabolism (Miyamoto et al., 2007; Lundin et al., 2011), and downregulated circuits between cortical and subcortical structures, which may be related to the occurrence of clinical symptoms.

Many previous studies (Calamante, 2010; Ziegelitz et al., 2014, 2015) assumed that occipital cortex perfusion was less affected by diseases and used the occipital lobe as an internal reference to evaluate the perfusion in other brain regions. However, the results of this study showed that the occipital lobe perfusion in patients with INPH was significantly reduced compared with that in HCs, suggesting that there may be some deviation between the assumptions of previous studies and the real situation.

In this study, there was a significant correlation between the baseline CBF in the frontal lobe, temporal lobe, basal ganglia, thalamus, and cingulate gyrus and poor gait manifestation. Elderly individuals have more brain regions involved in motor control than younger ones. Previous studies found that the CBF in the frontal periventricular white matter was significantly correlated with gait (Jurcoane et al., 2014). The prefrontal cortex, which receives almost all information from the sensory system and is preferentially connected to motor information processing structures, plays a core role in cognitive control of motor performance, and therefore, elderly individuals are more dependent on activation of the bilateral frontal cortex during exercise. The frontal periventricular corticobasal ganglia-thalamocortical pathways are constituted by the fibers of the frontal lateral ventricle connected with the supplementary motor cortex, basal ganglia, and thalamus, which are involved in gait and body balance control (Virhammar et al., 2014). The thalamus and cingulate gyrus play important roles in this pathway, so reduced local perfusion may lead to downregulation of pathway function, which can manifest as motor dysfunction (Ziegelitz et al., 2015). The thalamus is the main structure regulating basal ganglia function and plays an important role in motor function. These structures influence each other and contribute to the occurrence and outcome of gait disorders in the course of diseases.

In the study, preoperative CBF in the frontal lobe and basal ganglia was positively correlated with the severity of preoperative cognitive impairment associated with INPH and negatively correlated with the change of postoperative MMSE score. Decreased perfusion and metabolic disturbance lead to impaired ventricular white matter and then influence the function of the frontal-subcortical pathways and the frontal periventricular corticobasal ganglia-thalamocortical pathways, which further affects cognitive function. Previous studies have proven that this circuit is positively correlated with cognitive and psychological scores (Ziegelitz et al., 2016).

The improvement in motor function after shunting was negatively correlated with preoperative perfusion in the parietal lobe, occipital lobe, and temporal lobe. The occipital lobe is connected to the precuneus and frontal and temporal lobes through the cingulate tract and is involved in the regulation of spatial relations and visual attention of body movement (Tanglay et al., 2021). Some studies have shown that parietal occipital white matter damage is associated with gait disorder (de Laat et al., 2011). We hypothesized that it may be possible to predict the recovery of motor function after shunting by the perfusion damage observed in the parietal and occipitotemporal lobes before surgery.

The results showed that there was a moderate positive correlation between reduced anterior cingulate perfusion and improved postoperative urination. The higher brain’s net effect on micturition is thought to be inhibitory. Moreover, the micturition reflex passes through the dorsolateral frontal cortex, anterior cingulate cortex, and hypothalamus (Tish and Geerling, 2020) as a part of the frontal-subcortical pathway and is closely related to the urinary control function. Preoperative perfusion in the cingulate gyrus being associated with recovery of bladder function is likely to predict the functional restoration of the descending cingulate pathway or cingulate cortex after shunt surgery.

Some studies have suggested that preoperative whole-brain CBF is related to clinical outcomes, and patients with lower CBF show clinical improvements after shunt surgery, indicating that preoperative CBF may contribute to predicting clinical outcomes after shunt surgery (Klinge et al., 2002). The slightly lower significance of our results compared with previous studies may be related to the small sample size and heterogeneity among the subjects, as well as differences in research methods and evaluation criteria of clinical symptoms. The results of previous studies have been discrepant, and whether baseline CBF can predict the outcome of shunt surgery has not been determined. Therefore, it is necessary to expand the sample size and conduct more in-depth research on this issue.

In some patients, motor function significantly improved in the short term after shunt surgery, but the duration was short, with the shortest maintenance of nearly a week. Specific analysis of individual patients revealed that a long course of the disease was a common feature. Combined with a CT perfusion (CTP) study (Ziegelitz et al., 2016), we speculated that massive CSF drainage could improve periventricular perfusion in the short term, but there might be permanent damage to white matter, which may interfere with the improvements in gray matter perfusion through the frontal periventricular corticobasal ganglia-thalamocortical pathways.

Positron emission tomography perfusion imaging, the only technique that can intrinsically quantify perfusion, is considered the gold standard in the cerebral perfusion evaluation with imaging (Mattoli et al., 2020). Previous studies have validated the CBF value of PASL against PET, proving that PASL has high repeatability in HCs and patients with AD (Xu et al., 2010). The advantage of the study lies in the quantitative analysis of CBF based on the whole-brain voxel level. On the one hand, this method directly analyzes the original data and does not involve *a priori* assumption of artificially defined ROI, so it is not subject to the subjective influence of researchers. On the other hand, the object of statistical analysis is each voxel in the CBF map, so the statistical result is not affected by volume. The spatial normalization process using the individuals’ structural phase enables a voxel-based statistical comparison of brain images with different morphologies.

Compared with previous studies that take the occipital cortex as an internal reference to delineate regions of interest (ROIs), this study solved the limitations of manually drawing ROIs and avoided the influence of anatomical artifacts and volume on research results, resulting in higher accuracy. Therefore, voxel-based analysis has the advantages of automaticity, comprehensiveness, objectivity, and repeatability.

There were some limitations to this study that need to be considered. First, the post-labeling delay in the PASL technique used in this study was short (1,290 ms), which led to hypoperfusion artifacts in some subjects. Second, a single TI PASL was used in this study, which is less efficient and more dependent on model assumptions for the arterial transit time, resulting in slightly poorer accuracy. Considering that the subjects involved in this study could not undergo a long-time MRI scan due to cognitive impairment and old age, we chose the short post-labeling delay single TI PASL sequence as a trade-off. In the following study, we will continue to increase the sample size of the INPH group and strengthen follow-up, make a regression analysis of preoperative perfusion and clinical symptoms, and try to establish a prediction model of shunt efficacy to predict the efficacy through preoperative perfusion and help patients, their families, and healthcare professionals involved in treating INPH.

## Conclusion

In this study, we measured brain perfusion in the patients with diagnosed INPH before and 1 month after shunt surgery to investigate the relationship between preoperative CBF and postoperative CBF changes and clinical symptoms. The current findings suggest that the HCs and the patients with diagnosed INPH exhibited CBF differences in the whole cerebrum, especially in the high convexity, temporal gyrus, and frontal white matter. The perfusion in different brain regions in the patients with INPH was correlated with clinical symptoms, and improvements in clinical symptoms after shunting were affected by the preoperative CBF. This study demonstrates that widely distributed and intercorrelated cortical and subcortical pathways are involved in the development of INPH symptoms. The pathogenesis of hypoperfusion and its specific effects on disease development need to be further explored in combination with other imaging techniques and molecular studies.

## Data Availability Statement

The raw data supporting the conclusions of this article will be made available by the authors, without undue reservation.

## Ethics Statement

The studies involving human participants were reviewed and approved by the Institutional Review Board of Huadong Hospital affiliated with Fudan University. Written informed consent for participation was not required for this study in accordance with the national legislation and the institutional requirements.

## Author Contributions

WH, XF, SL, RM, CY, WL, and GL made a substantial contribution to the concept and design, acquisition of data or analysis, and interpretation of data. WH, XF, and SL drafted the manuscript and revised it critically for relevant intellectual content. WH and XF performed the MR examination and follow-up of patients. All authors made a substantial contribution to the concept and design, acquisition of data or analysis, interpretation of data, and approved the final version of the manuscript.

## Conflict of Interest

The authors declare that the research was conducted in the absence of any commercial or financial relationships that could be construed as a potential conflict of interest.

## Publisher’s Note

All claims expressed in this article are solely those of the authors and do not necessarily represent those of their affiliated organizations, or those of the publisher, the editors and the reviewers. Any product that may be evaluated in this article, or claim that may be made by its manufacturer, is not guaranteed or endorsed by the publisher.

## Footnotes

1
http://www.fil.ion.ucl.ac.uk/spm

2
https://cfn.upenn.edu/zewang/ASLtbx.php

3
http://www.alivelearn.net/xjview

## References

[B1] Adams R. D. Fisher C. M. Hakim S. Ojemann R. G. Sweet W. H. (1965). Symptomatic Occult Hydrocephalus With “Normal” Cerebrospinal-Fluid Pressure.A Treatable Syndrome. *New England J. Med.* 273 117–126. 10.1056/nejm196507152730301 14303656

[B2] Agerskov S. Hellström P. Andrén K. Kollén L. Wikkelsö C. Tullberg M. (2018). The phenotype of idiopathic normal pressure hydrocephalus-a single center study of 429 patients. *J.Neurol. Sci.* 391 54–60. 10.1016/j.jns.2018.05.022 30103972

[B3] Allali G. Laidet M. Armand S. Assal F. (2018). Brain comorbidities in normal pressure hydrocephalus. *Eur. J. Neurol.* 25 542–548. 10.1111/ene.13543 29222955 PMC5947755

[B4] Ashburner J. (2007). A fast diffeomorphic image registration algorithm. *NeuroImage* 38 95–113. 10.1016/j.neuroimage.2007.07.007 17761438

[B5] Bräutigam K. Vakis A. Tsitsipanis C. (2019). Pathogenesis of idiopathic Normal Pressure Hydrocephalus: A review of knowledge. *J. Clin. Neurosci.* 61 10–13. 10.1016/j.jocn.2018.10.147 30409528

[B6] Broggi M. Redaelli V. Tringali G. Restelli F. Romito L. Schiavolin S. (2016). Normal Pressure Hydrocephalus and Parkinsonism: Preliminary Data on Neurosurgical and Neurological Treatment. *World Neurosurg.* 90 348–356. 10.1016/j.wneu.2016.03.004 26970480

[B7] Buxton R. B. Frank L. R. Wong E. C. Siewert B. Warach S. Edelman R. R. (1998). A general kinetic model for quantitative perfusion imaging with arterial spin labeling. *Magn. Reson. Med.* 40 383–396. 10.1002/mrm.1910400308 9727941

[B8] Calamante F. (2010). Perfusion MRI using dynamic-susceptibility contrast MRI: quantification issues in patient studies. *Top. Magn. Reson. Imaging* 21 75–85. 10.1097/RMR.0b013e31821e53f5 21613873

[B9] de Laat K. F. Tuladhar A. M. van Norden A. G. Norris D. G. Zwiers M. P. de Leeuw F. E. (2011). Loss of white matter integrity is associated with gait disorders in cerebral small vessel disease. *Brain* 134 73–83. 10.1093/brain/awq343 21156660

[B10] Experts consensus on diagnosis and treatment of normal pressure hydrocephalus in China. (2016). Experts consensus on diagnosis and treatment of normal pressure hydrocephalus in China. *Zhonghua Yi Xue Za Zhi* 96 1635–1638.

[B11] Giordan E. Palandri G. Lanzino G. Murad M. H. Elder B. D. (2018). Outcomes and complications of different surgical treatments for idiopathic normal pressure hydrocephalus: a systematic review and meta-analysis. *J. Neurosurg.* 1 1–13. 10.3171/2018.5.Jns1875 30497150

[B12] Göttler J. Preibisch C. Riederer I. Pasquini L. Alexopoulos P. Bohn K. P. (2019). Reduced blood oxygenation level dependent connectivity is related to hypoperfusion in Alzheimer’s disease. *J. Cereb. Blood Flow Metab.* 39 1314–1325. 10.1177/0271678x18759182 29431005 PMC6668525

[B13] Ishii K. (2020). Diagnostic imaging of dementia with Lewy bodies, frontotemporal lobar degeneration, and normal pressure hydrocephalus. *Jpn. J. Radiol.* 38 64–76. 10.1007/s11604-019-00881-9 31549279

[B14] Jezzard P. Chappell M. A. Okell T. W. (2018). Arterial spin labeling for the measurement of cerebral perfusion and angiography. *J.Cereb. Blood Flow Metab.* 38 603–626. 10.1177/0271678x17743240 29168667 PMC5888859

[B15] Jurcoane A. Keil F. Szelenyi A. Pfeilschifter W. Singer O. C. Hattingen E. (2014). Directional diffusion of corticospinal tract supports therapy decisions in idiopathic normal-pressure hydrocephalus. *Neuroradiology* 56 5–13. 10.1007/s00234-013-1289-8 24158631

[B16] Klinge P. Berding G. Brinker T. Schuhmann M. Weckesser E. Knapp W. H. (2002). The role of cerebral blood flow and cerebrovascular reserve capacity in the diagnosis of chronic hydrocephalus–a PET-study on 60 patients. *Acta Neurochir. Suppl.* 81 39–41. 10.1007/978-3-7091-6738-0_10 12168352

[B17] Lalou A. D. Czosnyka M. Donnelly J. Pickard J. D. Nabbanja E. Keong N. C. (2018). Cerebral autoregulation, cerebrospinal fluid outflow resistance, and outcome following cerebrospinal fluid diversion in normal pressure hydrocephalus. *J. Neurosurg.* 130 154–162. 10.3171/2017.7.Jns17216 29547089

[B18] Landau S. M. Thomas B. A. Thurfjell L. Schmidt M. Margolin R. Mintun M. (2014). Amyloid PET imaging in Alzheimer’s disease: a comparison of three radiotracers. *Eur. J. Nucl. Med. Mol. Imaging* 41 1398–1407. 10.1007/s00259-014-2753-3 24647577 PMC4055504

[B19] Lundin F. Tisell A. Dahlqvist Leinhard O. Tullberg M. Wikkelsö C. Lundberg P. (2011). Reduced thalamic N-acetylaspartate in idiopathic normal pressure hydrocephalus: a controlled 1H-magnetic resonance spectroscopy study of frontal deep white matter and the thalamus using absolute quantification. *J. Neurol.Neurosurg.Psychiat.* 82 772–778. 10.1136/jnnp.2010.223529 21217158

[B20] Macki M. Mahajan A. Shatz R. Air E. L. Novikova M. Fakih M. (2020). Prevalence of Alternative Diagnoses and Implications for Management in Idiopathic Normal Pressure Hydrocephalus Patients. *Neurosurgery* 87 999–1007. 10.1093/neuros/nyaa199 32472677

[B21] Mattoli M. V. Treglia G. Calcagni M. L. Mangiola A. Anile C. Trevisi G. (2020). Usefulness of Brain Positron Emission Tomography with Different Tracers in the Evaluation of Patients with Idiopathic Normal Pressure Hydrocephalous. *Int. J. Mol. Sci.* 21:6523. 10.3390/ijms21186523 32906629 PMC7555923

[B22] Miyamoto J. Imahori Y. Mineura K. (2007). Cerebral oxygen metabolism in idiopathic-normal pressure hydrocephalus. *Neurol. Res.* 29 830–834. 10.1179/016164107X181851 17716389

[B23] Nakajima M. Yamada S. Miyajima M. Ishii K. Kuriyama N. Kazui H. (2021). Guidelines for Management of Idiopathic Normal Pressure Hydrocephalus (Third Edition): Endorsed by the Japanese Society of Normal Pressure Hydrocephalus. *Neurol. Med.Chir.* 61 63–97. 10.2176/nmc.st.2020-0292 33455998 PMC7905302

[B24] Relkin N. Marmarou A. Klinge P. Bergsneider M. Black P. M. (2005). Diagnosing idiopathic normal-pressure hydrocephalus. *Neurosurgery* 57 S4–S16. 10.1227/01.neu.0000168185.29659.c5 16160425

[B25] Soldozy S. Galindo J. Snyder H. Ali Y. Norat P. Yaǧmurlu K. (2019). Clinical utility of arterial spin labeling imaging in disorders of the nervous system. *Neurosurg. Focus* 47:E5. 10.3171/2019.9.Focus19567 31786550

[B26] Tanglay O. Young I. M. Dadario N. B. Briggs R. G. Fonseka R. D. Dhanaraj V. (2021). Anatomy and white-matter connections of the precuneus. *Brain Imaging Behav.* [Epub Online ahead of print]. 10.1007/s11682-021-00529-1 34448064

[B27] Tish M. M. Geerling J. C. (2020). The Brain and the Bladder: Forebrain Control of Urinary (In)Continence. *Front. Physiol.* 11:658. 10.3389/fphys.2020.00658 32719609 PMC7349519

[B28] Tuniz F. Vescovi M. C. Bagatto D. Drigo D. De Colle M. C. Maieron M. (2017). The role of perfusion and diffusion MRI in the assessment of patients affected by probable idiopathic normal pressure hydrocephalus. A cohort-prospective preliminary study. *Fluids Barriers CNS* 14:24. 10.1186/s12987-017-0072-3 28899431 PMC5596479

[B29] Virhammar J. Laurell K. Ahlgren A. Cesarini K. G. Larsson E. M. (2014). Idiopathic normal pressure hydrocephalus: cerebral perfusion measured with pCASL before and repeatedly after CSF removal. *J. Cereb. Blood Flow Metab.* 34 1771–1778. 10.1038/jcbfm.2014.138 25138210 PMC4269752

[B30] Virhammar J. Laurell K. Ahlgren A. Larsson E. M. (2017). Arterial Spin-Labeling Perfusion MR Imaging Demonstrates Regional CBF Decrease in Idiopathic Normal Pressure Hydrocephalus. *AJNR Am. J. Neuroradiol.* 38 2081–2088. 10.3174/ajnr.A5347 28860216 PMC7963573

[B31] Wang Z. Aguirre G. K. Rao H. Wang J. Fernández-Seara M. A. Childress A. R. (2008). Empirical optimization of ASL data analysis using an ASL data processing toolbox: ASLtbx. *Mag. Reson. Imaging* 26 261–269. 10.1016/j.mri.2007.07.003 17826940 PMC2268990

[B32] Xu G. Rowley H. A. Wu G. Alsop D. C. Shankaranarayanan A. Dowling M. (2010). Reliability and precision of pseudo-continuous arterial spin labeling perfusion MRI on 3.0 T and comparison with 15O-water PET in elderly subjects at risk for Alzheimer’s disease. *NMR Biomed.* 23 286–293. 10.1002/nbm.1462 19953503 PMC2843795

[B33] Ziegelitz D. Arvidsson J. Hellström P. Tullberg M. Wikkelsø C. Starck G. (2015). In Patients With Idiopathic Normal Pressure Hydrocephalus Postoperative Cerebral Perfusion Changes Measured by Dynamic Susceptibility Contrast Magnetic Resonance Imaging Correlate With Clinical Improvement. *J. Comp. Assist. Tomogr.* 39 531–540. 10.1097/rct.0000000000000254 25974719

[B34] Ziegelitz D. Arvidsson J. Hellström P. Tullberg M. Wikkelsø C. Starck G. (2016). Pre-and postoperative cerebral blood flow changes in patients with idiopathic normal pressure hydrocephalus measured by computed tomography (CT)-perfusion. *J. Cereb. Blood Flow Metab.* 36 1755–1766. 10.1177/0271678x15608521 26661191 PMC5076781

[B35] Ziegelitz D. Starck G. Kristiansen D. Jakobsson M. Hultenmo M. Mikkelsen I. K. (2014). Cerebral perfusion measured by dynamic susceptibility contrast MRI is reduced in patients with idiopathic normal pressure hydrocephalus. *J. Magn. Reson. Imaging* 39 1533–1542. 10.1002/jmri.24292 24006249

